# Teaching Strategies in Interventional Radiology: A Narrative Review of the Literature

**DOI:** 10.1007/s00270-024-03891-x

**Published:** 2024-10-26

**Authors:** Divya Srinivasan, Yakup Kilic, Gina K. Weston-Petrides, Rakesh Patel, Anosha Yazdabadi, Hamed Asadi, Roberto Luigi Cazzato, Behnam Shaygi

**Affiliations:** 1 Imperial College School of Medicine (ICSM), South Kensington Campus, London, SW7 2AZ UK; 2 UCLH, London, UK; 3 King’s College Hospital NHS Foundation Trust, London, UK; 4 London North West University Healthcare NHS Trust, The Hillingdon Hospitals NHS Foundation Trust, Honorary Clinical Reader, Brunel University London, London, UK; 5 Eastern Health Clinical School, Monash University and Eastern Health, Melbourne, Australia; 6 NeuroInterventional Radiology Unit, Monash Health, Melbourne, Australia; 7 School of Medicine, Deakin University, Waurn Ponds, Geelong, Australia; 8 Medicine, Nursing and Health Sciences, Monash University, Melbourne, Australia; 9 The Florey Institute of Neuroscience and Mental Health, Parkville, Australia; 10 Department Interventional Radiology, University Hospital of Strasbourg, Strasbourg, France; 11 Interventional and Diagnostic Radiologist, London North West University Healthcare NHS Trust, London, UK

**Keywords:** Education, Teaching, Simulation, Interventional radiology

## Abstract

**Introduction:**

Interventional radiology (IR) is a rapidly developing speciality where innovation—especially in teaching practices—is vital. With workforce and capacity shortages, synthesis of classical educational theories and novel strategies utilising virtual reality (VR) and artificial intelligence (AI) provide opportunities to make teaching as efficient and effective as possible. The aim of this review is to examine the literature on different approaches in IR teaching and learning in undergraduates and postgraduates.

**Methods:**

Literature was reviewed using a comprehensive search strategy with relevant keywords. Articles were limited to 2013–2023. Databases searched included MEDLINE, Embase, British Education Index and ERIC, in addition to a manual review of references.

**Results:**

Of the 2903 unique abstracts reviewed by the authors, 43 were relevant to the purpose of this study. The major pedagogical approaches identified were categorised into the following—traditional master-apprentice mentoring, virtual reality/simulation, physical models, and remote teaching. VR simulations enable practise free from the limits of time and risk to patients, as well as potential for standardised formal curricula. AI has the capability to enhance training simulations and assessment of trainees. With recent events necessitating innovation in online remote teaching, programs that are accessible whilst arguably imparting just as much clinical knowledge as in-person education have now been developed.

**Conclusion:**

Mentoring has conventionally been the standard for radiology teaching, however there are now several alternative pedagogical approaches available to the IR community. A combination of the most effective ideas within each is the optimal method by which IR should be taught.

## Introduction

Interventional radiology (IR) is a rapidly developing sub-specialty within radiology. Studies have demonstrated the critical role of IR in helping reduce morbidity, mortality and in-hospital stay through development of revolutionary treatment options [1]. The current growth in demand for IR procedures is widely acknowledged, alongside concomitant increase in number, scope, and complexity of procedures. However, in the United Kingdom (UK), currently there are not enough fully trained interventional radiologists to meet this demand [2].

It is well established within the medical school curriculum that IR is underrepresented with recent studies suggesting inadequate awareness of IR among medical students [3]. Consequently, students early on are not exposed to IR to consider it as a viable career, which in turn exacerbates the shortage of IR trainees in the UK. This underrepresentation of IR in the undergraduate curriculum is also seen in other developed countries such as in Canada, where 91% of students in multiple medical schools reported a lack of teaching or exposure in radiology as a whole [3][3].

Awareness and effective training in IR may help address these workforce shortages. Reviews suggest that implementation of didactic IR courses within one curriculum improves IR awareness and knowledge among students [5].

There is a lack of description of the ideal way to teach IR in an undergraduate setting in the literature. Novel strategies utilizing artificial intelligence (AI) and virtual reality (VR) have been recently adopted providing opportunities to advance educational practices, both alone and in conjunction with current methods.

## Aims

The aim of this qualitative review is to examine the existing literature exploring different approaches in how IR is currently taught in undergraduate and postgraduate settings, discussing current shortcomings and potential solutions.

## Methods

A review of the literature was performed using a comprehensive search focusing on IR teaching among medical students and radiology trainees.

Ethical approval was unnecessary as no primary data was involved.

### Identification and Selection of Articles

A search of publications was conducted in the databases MEDLINE, Embase, British Education Index and Institute of Education Sciences (ERIC), in addition to a manual review of references.

The following MeSH descriptors and respective Boolean operators were used to search for studies: *interventional radiology* OR *interventional oncology* OR *image guided surgery* OR *image guided procedure* or *endovascular surgery* AND *teaching* OR *medical education* OR *curriculum* OR *training*.

The inclusion criteria comprised peer-reviewed studies, reviews, and editorials published in English between 2013 and 2023, excluding grey literature. Articles were included if they focused on pedagogical approaches in IR in undergraduate and postgraduate settings, and assessed the effectiveness or limitations of approaches. Articles were excluded if they addressed medical specialties other than IR, non-medical applications, or involved non-human subjects.

The studies identified via this process were inserted into a standardised table for data extraction and analysis, and duplicate studies were removed.

**Fig. 1:**
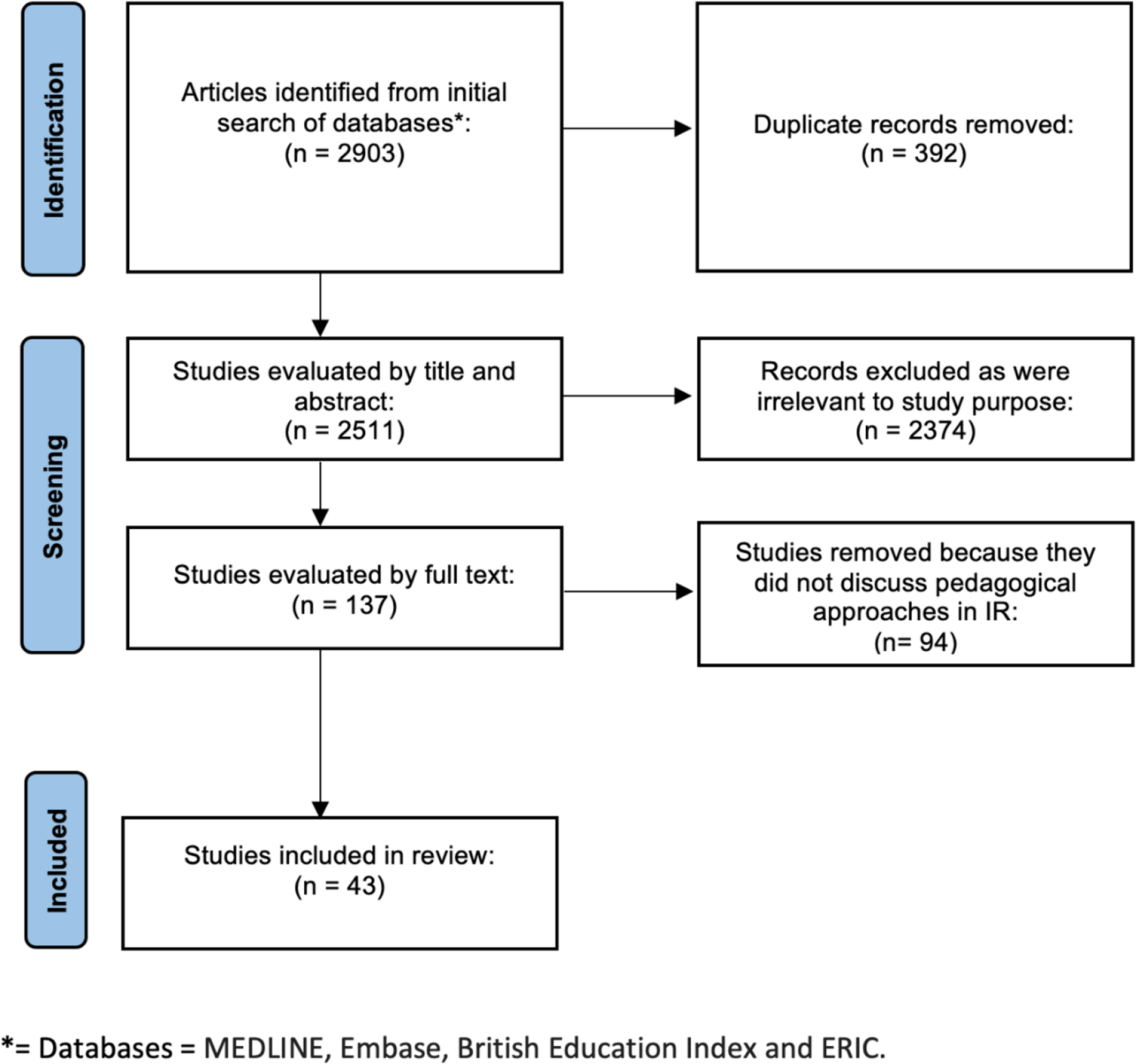
PRISMA flow diagram showing the identification, screening, and selection stages in reviewing studies on pedagogical approaches in Interventional radiology

Figure 1 illustrates the article selection process.

### Data Synthesis and Analysis

Information extracted from the articles included the type and year of publication, methodology used and measured efficacy of pedagogical approach. The major pedagogical approaches identified were categorized by approach.

## Results

The initial article searches generated 2903 studies. Of these, 392 duplicate records were removed. The remaining unique article abstracts and/or titles were reviewed by the author. 2374 articles were removed after screening of title and abstract as they did not fit the inclusion criteria. Following full text evaluation, a further 94 articles were removed. Finally, 43 were found to be pertinent to the purpose of exploring pedagogy of IR and were included in this review.

### Study Characteristics

Of the 43 eligible articles, 30 were categorised as descriptive studies, four as systematic reviews, three as literature reviews, two as randomised control trials (RCT) and four as editorials or expert opinions. Of the studies, 28 were prospective and two retrospective in nature.

### Pedagogical Approaches Found

The major pedagogical approaches identified from these articles were categorised into; traditional master-apprentice mentoring, VR/simulation, physical models, and remote teaching.

The Master-apprentice approach was cited most as the standard method of teaching, however in the literature there was much interest in novel teaching methods, as indicated by the greater number of studies identified.

The number of papers included in this review by teaching method are shown below in Table 1.

**Table 1:** Teaching approach reported by selected articles

Teaching approach	No. papers mentioned
Master-apprentice mentoring	6
Physical models	17
Virtual reality (VR) and Simulation	15
Remote and online teaching	5

### Traditional Master-Apprentice Mentoring

Mentorship is currently the standard pillar of IR training, especially crucial in IR research [6, 7]. Effective IR mentors are a source of guidance, personal and career inspiration, and institutional support. Studies found that trainees with an IR mentor were significantly more likely to pursue research, publish more papers, and have greater overall career satisfaction [8].

However, current pressures on healthcare systems including cost concerns and capacity limitations, have led to reduced dedicated teaching time for trainees. Among undergraduates especially, reviews of the current system found a significant gap in IR teaching opportunities, the limited exposure likely contributing to the low recruitment and interest in the specialty [9]. A study conducted across centers around the world found lack of dedicated teaching time and structured support to be the greatest limitations of the current teaching model [4].

Another major disadvantage in this approach is the discrepancy in experience between female and male trainees. Female IR residents reported significantly greater difficulty in finding mentors, were significantly less likely to feel valued in research efforts and subjectively received less IR mentorship [10]. Promotion of gender equality in mentorship is therefore a highly necessary improvement. One study suggests that earlier exposure among medical students is an effective way to reduce gender-based biases among prospective trainees [11].

### Physical Models

Physical models enable deliberate practice that can be repeated multiple times for mastery learning in a safe learning environment. There is evidence that anatomical models for ultrasound guided central venous catheter placement improve learner proficiency and confidence [12]. A recent randomised control trial found that training on models translate into a significantly improved first attempt success rate and needle-tip positioning in patient procedures [13].

Mastery of thoracentesis on simulators increased confidence in learners, corresponding with less referrals and more bedside procedures [14]. Fluoroscopically guided lumbar puncture phantoms led to significantly lower procedural time, and thus reduced patient radiation dose compared to those traditionally taught [15].

Moreover, models can be made with a variety of different materials and methods.

Fresh frozen cadavers provide realistic models with high vascular patency, allowing 100% success rates in image-guided artery cannulation, with one specimen being suitable for reuse a maximal of four times [16]. Studies report better construct validity than VR simulators for endovascular training use, with haptic feedback from cadavers reportedly better than the “artificial” feeling VR simulator [17, 18]. The thiel cadaveric model incorporated pulsatile flow to successfully model aortic perfusion for endovascular aortic repair procedures [19]. Although cadavers can be a cost-effective simulation method, limitations such as ethics and availability exist. Interestingly, human placentas were explored as a less resource intensive model for angiography and stent placement—with face and construct validity confirmed to be sufficient for education [20].

Animal models also have the advantage of a constant blood flow and hemodynamic forces. Porcine in-vivo bile duct dilation models were validated to be a realistic training model with full animal survival and low complication rates [21]. Porcine liver tumor models provide valuable image-guided procedural practice both in vivo and ex vivo [22]. However, the anatomy differs from humans and similarly ethics must be considered.

Custom 3-dimensional (3D) printed models offer the possibility of creating patient-specific conditions e.g., stenosis, enabling rehearsal of challenging cases. Use of a custom femoral artery vascular access model significantly improved student confidence as compared to trainees using a standard commercial vascular access model [23]. Patient specific thoracic-spine models were developed for thoracic epidural analgesia, with higher success rates in real-life procedures in those trained on this model versus without [24]. A 3D printed simulator for the challenging percutaneous transhepatic cholangiography and drainage procedure significantly increased trainee’s speed and confidence [25]. One systematic review identifies a major limitation of 3D printing to be cost but proposes this be mitigated by sharing costs with other departments like cardiology, as models can be designed to be repeatedly used for different procedures [26].

On the other hand low-cost simulations can be made with inexpensive materials. Gelatin, silicone and plastic can be used, but are time-consuming to produce and have variable stiffness [27]. These models are most useful for teaching trainees earlier on in their career for techniques in needle manipulation [28]. High fidelity need not be the only goal, as low fidelity models still improve knowledge and confidence of trainees after Computer tomography (CT) guided procedures as compared to standard didactic learning [29].

### Simulation and Virtual Reality (VR)

Simulation can also be an effective assessment tool, as an angiography simulator study found the simulator to accurately distinguish skill differences between IR beginners and experts when tested [30]. In addition, simulation offers an avenue to create a standardized and structured curriculum [1].

However, the over-arching limitation of simulation is that even the most realistic ones are not as valuable as real-life patient experience which is deemed gold-standard in IR curricula [31].

VR uses computerised technology to realistically immerse a user in a particular situation and remove the actual environment [32]. Augmented reality (AR) allows superimposition of computer-generated elements onto real world environments [33]. Superimposition offers an alternative to operators alternating gaze between the interventional field and a different instrumentation screen.

V/AR has been used in IR since 1996, with simulation of procedures and scenarios shown to improve technical skills and competence in several studies [34, 35]. Several high-fidelity computer-based simulators are currently available, for example the ‘ANGIO-Mentor’, which has a realistic interface, and option to mimic complication scenarios such as dissections [36]. Procedural time and number of errors for endovascular aneurysm repair significantly decreased in trainees post Simbionix EVAR simulator [37]. Studies also propose the potential for these computerised simulators to provide a personalized curriculum adaptable to trainee needs, where common errors are focused on.

In the current climate of workforce limitations, feedback can be scarce. The advantage of VR is that it can give detailed automated results such as time-action analysis. A fluoroscopy guided lumbar puncture model provided excellent haptic feedback and validity, while also providing useful data on distance to target, number of fluoroscopic shots etc. [38]. Another advantage is that the computerised nature allows for the practice of rare or difficult cases that seldom naturally present in clinical practice to be programmed in.

VR simulation not only has value in procedural practice, but clinical scenarios can also be practiced. High acuity simulation for a contrast reaction scenario improved knowledge and confidence among IR residents [39]. Recently, an immersive simulator that enables assessment in carotid artery stenting of an entire endovascular team, has been developed, although this is associated with much space and maintenance requirements [40]. Patient specific VR assisted “whole-team rehearsal” simulations have been shown to reduce errors and surgical time as compared to no warm-up sessions [41]. This is especially useful, as imperfections in teamwork and communication reportedly contribute to the highest errors in endovascular procedures [42].

Simulation also allows undergraduates, who would not be able to carry out such technical IR procedures themselves, to gain exposure of procedures such as carotid stenting. This early introduction of IR into undergraduate curricula is likely to increase interest in IR, a specialty that has traditionally been underrepresented in medical school. A VR IR elective implemented in first and second year medical students successfully increased exposure and understanding of the sub-specialty [43]. An endovascular simulator significantly improved likelihood of students to pursue IR, and “attractiveness” of the specialty, indicating that utilization of VR can potentially mitigate the recruitment problem IR is currently facing [44].

IR is a specialty with global disparity in its accessibility due to the cost-prohibitive nature of the required equipment [1]. Therefore, strategies like VR that do not require IR centers physically carrying out these procedures could potentially mitigate this locational disparity in IR education [45].

A major reported limitation of VR is its accessibility and usability issue, as many models are large. However not all simulators need to be large, for example a study investigating the development of VR glasses for central line placement improved procedural time and adherence to the Centre for disease control (CDC) guidelines, while reportedly being easy and convenient to use [46].

The most realistic simulations often involve state-of-the-art haptics. This requires the collation of performance metrics and validation which corresponds with increased cost and time invested to its development. One study proposes that a VR simulation cannot be purchased for under £100,000 [34].

However, some argue that VR is still less expensive than training in an operating theatre. A randomized control trial cost analysis found that VR based training cost €1195 less than conventional angio-suite training for endovascular procedure proficiency, even without considering costs related to potential complications [47].

Additionally, meta-analysis has found no significant association between fidelity of simulators and performance metrics [48].

### Remote and Online Teaching

The impact of the coronavirus pandemic on all aspects of medical education has highlighted the value of remote teaching to continue to ensure the quality of education is maintained. In residents preparing for core exams, implementation of a virtual IR course was found to significantly improve participants confidence and was preferred over in-person learning [49]. Webinar-based IR lectures have been shown to have positive impacts on improving interest in IR among undergraduates, as compared to the scarce in-person IR rotations [50]. The “flipped-classroom”, interactive virtual lesson approach was ranked to have the highest educational value in medical students undergoing a developed IR course, with significant increases in knowledge of IR procedures and roles post course [51]. Networking is crucial to IR research, and greater use of remote communication could help tackle limitations of widening access in IR, as the remote nature allows global engagement [52].

Remote guidance from specialists could also be utilized in training. AR in conjunction with tele-proctorship, has been explored with a Proximie® platform. Specialists can demonstrate and annotate with the integrated AI system, while operators work, with operators reporting satisfactory levels of safety [53].

The overarching limitation of remote teaching is that IR is a hands-on learning discipline. Augmentation with live/recorded cases are not sufficient to fulfill all needs of procedural training.

## Discussion

The predominant way IR is taught is through the traditional master-apprentice model. However, recent literature brings to light the shortcomings of this approach in keeping up with the modern demands of the speciality. The main issues identified by this review are as follows:

1. Medical student engagement is limited, hindering trainee recruitment.
2. Increased demand for IR procedures diverts resources from training to service delivery, limiting support for trainee positions.
3. Participation in mentor/mentee training is limited by the need to physically be in the training environment.

Consequentially, there is a call for educational practices to evolve from the traditional model to provide enough well-trained interventional radiologists to meet healthcare demands.

This evolution likely involves incorporating methods such as V/AR simulation into a blended curriculum. Blended education with online and on-site hands-on training was found to be both low cost and preferred by postgraduates in a pilot study [54]. An RCT investigating an endovascular curriculum incorporating online learning and VR simulation and multiple other studies found trainees completing the virtual programs demonstrated superior technical endovascular skills, as compared to peers with standard education [55–58]. Systematic review suggests that optimal use of VR in IR training is in conjunction with traditional modes [59].

In other catheter-based medical specialties, e.g., vascular surgery, simulation has been accepted as an important tool, and similarly IR could benefit from making it a mainstay in the training curriculum [60]. The World Federation of Interventional Neuroradiologists highly recommended simulation for basic training early in careers, and encourages development of new tools for more advanced neuro-interventions [61]. Many predict that virtual approaches will take the foreground as technology advances [62].

While, simulator training is promising, the evidence is limited by the small-scale nature of these pilot trials. There is a gap in the literature detailing whether patient outcomes were improved. To justify shifting the educational paradigm, simulators must be incorporated into larger programs, where robust research on its benefits can be conducted [63]. Remote proctorship could be a useful way to enable specialists to take part in the hands-on learning of IR in areas trainees would not routinely have access to such specialists. As a proof-of-concept case, the aforementioned Proximie® system enabled an experienced surgeon to guide a reconstructive surgery on a hand in Gaza [64]. Rural surgeons can utilise telemonitoring, as surveys demonstrate this group found telemonitoring would be useful for skill acquisition [65]. With this system expertise can be shared from around the world using any device and data from procedures can be securely captured and analyzed.

Furthermore, the role of AI in medicine is rapidly emerging. Combining AI with AR technology could yield a novel method of training and trainee assessment [66]. Among trainees, there is an overall positive attitude of radiologists towards learning more about AI in radiology education [67].

### Limitations

This review was limited to articles available in the English Language and excluded grey literature, so does not encompass all literature worldwide. Furthermore, the search terms may not identify all relevant articles.

The majority of studies included used small sample sizes and were single-institution based research products—limiting the generalisation of these results.

## Conclusion

Interventional Radiologists have minimal formal training in the delivery of IR Education specifically, with limited focus given to educational science and latest advancements in the educational field. To meet the increasing demands on the specialty, there is a call to reflect on current teaching practices and incorporate more scientific and novel pedagogical methods. Curriculums that blend V/AR, simulation, and remote aspects of teaching, especially in early training stages, show promise in the literature thus far. However, evidence is still evolving with many unexplored avenues. With further larger studies and greater investment in educational science research, we will gain the insight and evidence needed to best address these educational demands.

## References

[1] European Society of Radiology (2018) Summary of the proceedings of the International Forum 2017 Position of interventional radiology within radiology. Insights Imaging 9(2): 189–97 10.1007/s13244-018-0594-5 PMC5893490 29476428

[2] Radiologists TRCoRaBSoi. Investing in the interventional radiology workforce: the quality and efficacy case. The Royal College of Radiologists. 2014

[3] Dmytriw AA, Mok PS, Gorelik N, Kavanaugh J, Brown P. Radiology in the undergraduate medical curriculum: too little, too late? Med Sci Educ. 2015;25(3):223–7. 28286696 10.1007/s40670-015-0130-x PMC5325049

[4] Makris GC, Burrows V, Lyall F, Moore A, Hamady M. Vascular and interventional radiology training; international perspectives and challenges. Cardiovasc Intervent Radiol. 2020;44(3):462–72. 33174143 10.1007/s00270-020-02688-y

[5] Seyyed MH, Parham TB, Davood D, Reza M, Seyed AG, Masoumeh G. Worldwide knowledge about interventional radiology among medical students: findings of a comprehensive review. Cardiovasc Intervent Radiol. 2023;46(11):1641–54. 37798431 10.1007/s00270-023-03558-z

[6] Cressman ENK, Newton I, Larson AC, Woodrum DA, Srimathveeravalli G, Borrelli MJ, et al. State of the research enterprise in IR and recommendations for the future: proceedings from the society of interventional radiology foundation investigator development task force. J Vasc Interv Radiol. 2018;29(6):751–7. 29709441 10.1016/j.jvir.2018.02.009

[7] Retrouvey M, Grajo JR, Awan O, Catanzano T, Cheong LHA, Mankoff D, et al. Transitioning from radiology training to academic faculty: the importance of mentorship. Curr Probl Diagn Radiol. 2020;49(4):219–23. 30904346 10.1067/j.cpradiol.2019.02.011

[8] Ward EC, Hargrave C, Brown E, Halkett G, Hogg P. Achieving success in clinically based research: the importance of mentoring. J Med Radiat Sci. 2017;64(4):315–20. 28653426 10.1002/jmrs.234 PMC5715317

[9] Emin EI, Ruhomauly Z, Theodoulou I, Hanrahan JG, Staikoglou N, Nicolaides M, et al. Are interventional radiology and allied specialities neglected in undergraduate medical education? A systematic review. Ann Med Surg (Lond). 2019;40:22–30. 30962927 10.1016/j.amsu.2019.03.004 PMC6429536

[10] Li S, Sun VH, Galla N, Salazar G, Lewis T, Ahmed M, et al. Gender-based survey analysis of research and mentoring in interventional radiology. J Vasc Interv Radiol. 2022;33(5):578-85.e3. 35114399 10.1016/j.jvir.2022.01.010

[11] LE Kumar V, Diaz A, Vinson A, Conrad M, LaBerge J. Early exposure improves medical student perceptions on female and minority physician inclusion in interventional radiology. J Vasc Interv Radiol. 2017;28(2):22.

[12] Bayci AW, Mangla J, Jenkins CS, Ivascu FA, Robbins JM. Novel educational module for subclavian central venous catheter insertion using real-time ultrasound guidance. J Surg Educ. 2015;72(6):1217–23. 26481424 10.1016/j.jsurg.2015.07.010

[13] Oh EJ, Lee JH, Kwon EJ, Min JJ. Simulation-based training using a vessel phantom effectively improved first attempt success and dynamic needle-tip positioning ability for ultrasound-guided radial artery cannulation in real patients: an assessor-blinded randomized controlled study. PLoS ONE. 2020;15(6):e0234567. 32525955 10.1371/journal.pone.0234567 PMC7289374

[14] Barsuk JH, Cohen ER, Williams MV, Scher J, Feinglass J, McGaghie WC, et al. The effect of simulation-based mastery learning on thoracentesis referral patterns. J Hosp Med. 2016;11(11):792–5. 27273066 10.1002/jhm.2623

[15] Faulkner AR, Bourgeois AC, Bradley YC, Hudson KB, Heidel RE, Pasciak AS. Simulation-based educational curriculum for fluoroscopically guided lumbar puncture improves operator confidence and reduces patient dose. Acad Radiol. 2015;22(5):668–73. 25863793 10.1016/j.acra.2014.12.024

[16] Jansen MM, Hazenberg C, de Ruiter QMB, van Hamersvelt RW, Bleys R, van Herwaarden JA. Feasibility of fresh frozen human cadavers as a research and training model for endovascular image guided interventions. PLoS ONE. 2020;15(11):e0242596. 33254200 10.1371/journal.pone.0242596 PMC7704126

[17] Nesbitt CI, Tingle SJ, Williams R, McCaslin JE, Searle R, Mafeld S, et al. Educational impact of a pulsatile human cadaver circulation model for endovascular training. Eur J Vasc Endovasc Surg. 2019;58(4):602–8. 31495728 10.1016/j.ejvs.2019.03.026

[18] Nesbitt C, Tingle SJ, Williams R, McCaslin J, Searle R, Mafeld S, et al. A pulsatile fresh frozen human cadaver circulation model for endovascular training: a trial of face validity. Ann Vasc Surg. 2018;46:345–50. 28887245 10.1016/j.avsg.2017.07.030

[19] McLeod H, Cox BF, Robertson J, Duncan R, Matthew S, Bhat R, et al. Human thiel-embalmed cadaveric aortic model with perfusion for endovascular intervention training and medical device evaluation. Cardiovasc Intervent Radiol. 2017;40(9):1454–60. 28451810 10.1007/s00270-017-1643-z PMC5541076

[20] Ribeiro de Oliveira MM, Nicolato A, Santos M, Godinho JV, Brito R, Alvarenga A, et al. Face, content, and construct validity of human placenta as a haptic training tool in neurointerventional surgery. J Neurosurg. 2016;124(5):1238–44. 26452122 10.3171/2015.1.JNS141583

[21] Gimenez ME, Garcia Vazquez A, Davrieux CF, Verde JM, Serra E, Palermo M, et al. Image-guided surgical training in percutaneous hepatobiliary procedures: development of a realistic and meaningful bile duct dilatation porcine model. J Laparoendosc Adv Surg Tech A. 2021;31(7):790–5. 32991240 10.1089/lap.2020.0680

[22] Garcia Vazquez A, Rodriguez-Luna MR, Verde J, Piantanida E, Alonci G, Palermo M, et al. Image-guided surgical simulation in minimally invasive liver procedures: development of a liver tumor porcine model using a multimodality imaging assessment. J Laparoendosc Adv Surg Tech A. 2021;31(10):1097–103. 34171972 10.1089/lap.2021.0105

[23] Sheu AY, Laidlaw GL, Fell JC, Triana BP, Goettl CS, Shah RP. Custom 3-dimensional printed ultrasound-compatible vascular access models: training medical students for vascular access. J Vasc Interv Radiol. 2019;30(6):922–7. 31126603 10.1016/j.jvir.2019.02.011

[24] Bortman J, Baribeau Y, Jeganathan J, Amador Y, Mahmood F, Shnider M, et al. Improving clinical proficiency using a 3-dimensionally printed and patient-specific thoracic spine model as a haptic task trainer. Reg Anesth Pain Med. 2018;43(8):819–24. 29894394 10.1097/AAP.0000000000000821

[25] Fechner C, Reyes del Castillo T, Roos JE, Zech CJ, Takes M, López BR. 3D printed percutaneous transhepatic cholangiography and drainage (PTCD) simulator for interventional radiology. Cardiovasc Intervent Radiol. 2023;46(1):1454–60. 10.1007/s00270-022-03347-0 36635370

[26] Tenewitz C, Le RT, Hernandez M, Baig S, Meyer TE. Systematic review of three-dimensional printing for simulation training of interventional radiology trainees. 3D Print Med. 2021;7(1):10. 33881672 10.1186/s41205-021-00102-y PMC8059217

[27] Zhao Z, Ma Y, Mushtaq A, Radhakrishnan V, Hu Y, Ren H, et al. Engineering functional and anthropomorphic models for surgical training in interventional radiology: a state-of-the-art review. Proc Inst Mech Eng H. 2023;237(1):3–17. 36377860 10.1177/09544119221135086 PMC9841824

[28] Nhan C, Chankowsky J, Torres C, Boucher LM. Creating low-cost phantoms for needle manipulation training in interventional radiology procedures. Radiographics. 2021;41(4):1230–42. 34048277 10.1148/rg.2021200133

[29] Picard M, Nelson R, Roebel J, Collins H, Anderson MB. Use of low-fidelity simulation laboratory training for teaching radiology residents CT-guided procedures. J Am Coll Radiol. 2016;13(11):1363–8. 27435881 10.1016/j.jacr.2016.05.025

[30] Jensen UJ, Jensen J, Olivecrona GK, Ahlberg G, Tornvall P. Technical skills assessment in a coronary angiography simulator for construct validation. Simul Healthc. 2013;8(5):324–8. 23598862 10.1097/SIH.0b013e31828fdedc

[31] Kallini JR, Makary MS, Patel S, Jang B, Kansagra K, Tew D, et al. The interventional radiology clinic teaching model: survey of ir residency programs. Cardiovasc Intervent Radiol. 2021;44(2):351–3. 33083856 10.1007/s00270-020-02672-6

[32] Brigham TJ. Reality check: basics of augmented, virtual, and mixed reality. Med Ref Serv Q. 2017;36(2):171–8. 28453428 10.1080/02763869.2017.1293987

[33] Mitha AP, Almekhlafi MA, Janjua MJ, Albuquerque FC, McDougall CG. Simulation and augmented reality in endovascular neurosurgery: lessons from aviation. Neurosurgery. 2013;72(Suppl 1):107–14. 23254798 10.1227/NEU.0b013e31827981fd

[34] Samadbeik M, Yaaghobi D, Bastani P, Abhari S, Rezaee R, Garavand A. The applications of virtual reality technology in medical groups teaching. J Adv Med Educ Prof. 2018;6(3):123–9. 30013996 PMC6039818

[35] Uppot RN, Laguna B, McCarthy CJ, De Novi G, Phelps A, Siegel E, et al. Implementing virtual and augmented reality tools for radiology education and training, communication, and clinical care. Radiology. 2019;291(3):570–80. 30990383 10.1148/radiol.2019182210

[36] Pannell JS, Santiago-Dieppa DR, Wali AR, Hirshman BR, Steinberg JA, Cheung VJ, et al. Simulator-based angiography and endovascular neurosurgery curriculum: a longitudinal evaluation of performance following simulator-based angiography training. Cureus. 2016;8(8):e756. 27733961 10.7759/cureus.756 PMC5045334

[37] Saratzis A, Calderbank T, Sidloff D, Bown MJ, Davies RS. Role of simulation in endovascular aneurysm repair (EVAR) training: a preliminary study. Eur J Vasc Endovasc Surg. 2017;53(2):193–8. 28003104 10.1016/j.ejvs.2016.11.016

[38] Ali S, Qandeel M, Ramakrishna R, Yang CW. Virtual simulation in enhancing procedural training for fluoroscopy-guided lumbar puncture: a pilot study. Acad Radiol. 2018;25(2):235–9. 29032887 10.1016/j.acra.2017.08.002

[39] Pfeifer K, Staib L, Arango J, Kirsch J, Arici M, Kappus L, et al. High-fidelity contrast reaction simulation training: performance comparison of faculty, fellows, and residents. J Am Coll Radiol. 2016;13(1):81–7. 26549266 10.1016/j.jacr.2015.08.016

[40] Lonn L, Edmond JJ, Marco J, Kearney PP, Gallagher AG. Virtual reality simulation training in a high-fidelity procedure suite: operator appraisal. J Vasc Interv Radiol. 2012;23(10):1361–6. 22854318 10.1016/j.jvir.2012.06.002

[41] Willaert WI, Aggarwal R, Daruwalla F, Van Herzeele I, Darzi AW, Vermassen FE, et al. Simulated procedure rehearsal is more effective than a preoperative generic warm-up for endovascular procedures. Ann Surg. 2012;255(6):1184–9. 22566016 10.1097/SLA.0b013e31824f9dbf

[42] Albayati MA, Gohel MS, Patel SR, Riga CV, Cheshire NJ, Bicknell CD. Identification of patient safety improvement targets in successful vascular and endovascular procedures: analysis of 251 hours of complex arterial surgery. Eur J Vasc Endovasc Surg. 2011;41(6):795–802. 21320788 10.1016/j.ejvs.2011.01.019

[43] Mills AC, Goldman DT, Marinelli BS, Sanghvi J, Garcia-Reyes K, Shilo D, et al. Leveraging the virtual learning environment to enhance medical student engagement with interventional radiology. Clin Imaging. 2023;96:26–30. 36738667 10.1016/j.clinimag.2023.01.007

[44] Stoehr F, Schotten S, Pitton MB, Dueber C, Schmidt F, Hansen NL, et al. Endovascular simulation training: a tool to increase enthusiasm for interventional radiology among medical students. Eur Radiol. 2020;30(8):4656–63. 32221683 10.1007/s00330-019-06646-2

[45] McKenney AS, Garg T, Kim E, Kesselman A. Addressing global radiology disparities: increasing access to interventional radiology education. Radiographics. 2021;41(5):E142–4. 34469223 10.1148/rg.2021210176

[46] Huang CY, Thomas JB, Alismail A, Cohen A, Almutairi W, Daher NS, et al. The use of augmented reality glasses in central line simulation: “see one, simulate many, do one competently, and teach everyone.” Adv Med Educ Pract. 2018;9:357–63. 29785148 10.2147/AMEP.S160704 PMC5953413

[47] Maertens H, Vermassen F, Aggarwal R, Doyen B, Desender L, Van Herzeele I, et al. Endovascular training using a simulation based curriculum is less expensive than training in the hybrid angiosuite. Eur J Vasc Endovasc Surg. 2018;56(4):583–90. 30131277 10.1016/j.ejvs.2018.07.011

[48] Patel R, Dennick R. Simulation based teaching in interventional radiology training: is it effective? Clin Radiol. 2017;72(3):266 e7-266 e14. 27986263 10.1016/j.crad.2016.10.014

[49] Shin DS, Greenberg CH, Woerner A, Monroe EJ, Hage AN, Bertino FJ, et al. Virtual interventional radiology education increases confidence in American board of radiology core exam preparation. Clin Imaging. 2023;95:90–1. 36682181 10.1016/j.clinimag.2022.12.013

[50] Kumar V, Szeto H, Lehrman ED, Kohlbrenner RM, Kolli PK, Wilson MW, et al. Expanding the teaching toolbox: characterizing utility of a web-based lecture series in educating future colleagues and referrers about the field of IR. J Vasc Interv Radiol. 2019;30(4):589–933. 30910181 10.1016/j.jvir.2018.11.010

[51] DePietro DM, Santucci SE, Harrison NE, Kiefer RM, Trerotola SO, Sudheendra D, et al. Medical student education during the COVID-19 pandemic: initial experiences implementing a virtual interventional radiology elective course. Acad Radiol. 2021;28(1):128–35. 33132008 10.1016/j.acra.2020.10.005 PMC7572083

[52] Ng HH, Chan VW, Zahid M, Ogunyanwo DAB, Stephens S, Jarosz D, et al. A global cross-sectional evaluation of teaching and perceptions of interventional radiology amongst undergraduate medical students and junior doctors and the role of a virtual interventional radiology symposium. Clin Radiol. 2021;76(12):935–7. 34602282 10.1016/j.crad.2021.09.007

[53] Patel S, Nourzaie R, Karunanithy N, Ilyas S, Gangi A, Diamantopoulos A. Remote proctorship: bringing world class expertise to every operating table. Cardiovasc Intervent Radiol. 2023;46(4):538–40. 36690818 10.1007/s00270-023-03364-7

[54] Neri EC, Crocetti L, Lorenzoni G, Cioni R, Brady A, Caramella D. Students opinion about E-learning in a master course in interventional radiology: a survey among participants. Digital Diagnostics. 2021. 10.17816/DD53701.

[55] Maertens H, Aggarwal R, Moreels N, Vermassen F, Van Herzeele I. A Proficiency Based Stepwise Endovascular Curricular Training (PROSPECT) Program Enhances Operative Performance in Real Life: A Randomised Controlled Trial. Eur J Vasc Endovasc Surg. 2017 Sep;54(3):387-396. 10.1016/j.ejvs.2017.06.011 28734705

[56] Theodoulou I, Louca C, Sideris M, Nicolaides M, Agrawal D, Halapas A, et al. A prospective study integrating a curriculum of interventional radiology in undergraduate education: a tetra-core simulation model. CVIR Endovasc. 2020;3(1):12. 32147761 10.1186/s42155-020-0104-y PMC7061096

[57] Sideris M, Hanrahan J, Tsoulfas G, Theodoulou I, Dhaif F, Papalois V, et al. Developing a novel international undergraduate surgical masterclass during a financial crisis: our 4-year experience. Postgrad Med J. 2018;94(1111):263–9. 29519810 10.1136/postgradmedj-2017-135479

[58] Feyen L, Minko P, Franke N, Volker M, Haage P, Paprottka P, et al. Feasibility of network-based, online endovascular simulator training in real time: results from a pilot study. Rofo. 2023;195(6):514–20. 36863363 10.1055/a-1994-7381

[59] Gelmini AYP, Duarte ML, de Assis AM, Guimarães Junior JB, Carnevale FC. Virtual reality in interventional radiology education: a systematic review. Radiol Bras. 2021;54:254–60. 34393293 10.1590/0100-3984.2020.0162 PMC8354189

[60] Mandal I, Ojha U. Training in interventional radiology: a simulation-based approach. J Med Educ Curric Dev. 2020;7:2382120520912744. 32313840 10.1177/2382120520912744 PMC7155237

[61] Picard L, Rodesch G, Bracard S, Taylor A. Recommendation of the WFITN regarding simulation in neurointerventional training. Interv Neuroradiol. 2017;23(3):237. 28335660 10.1177/1591019917696247 PMC5490866

[62] Avramov P, Avramov M, Jukovic M, Kadic V, Till V. Virtual simulation as a learning method in interventional radiology. Med Pregl. 2013;66(7–8):335–40. 24069818 10.2298/mpns1308335a

[63] Miller ZA, Amin A, Tu J, Echenique A, Winokur RS. Simulation-based training for interventional radiology and opportunities for improving the educational paradigm. Tech Vasc Interv Radiol. 2021;24(4):100764. 34895705 10.1016/j.tvir.2021.100764

[64] Greenfield MJ, Luck J, Billingsley ML, Heyes R, Smith OJ, Mosahebi A, et al. Demonstration of the effectiveness of augmented reality telesurgery in complex hand reconstruction in Gaza. Plast Reconstr Surg Glob Open. 2018;6(3):e1708. 29707463 10.1097/GOX.0000000000001708 PMC5908501

[65] Meek RD, Lungren MP, Gichoya JW. Machine learning for the interventional radiologist. AJR Am J Roentgenol. 2019;213(4):782–4. 31166764 10.2214/AJR.19.21527

[66] Holden MS, Xia S, Lia H, Keri Z, Bell C, Patterson L, et al. Machine learning methods for automated technical skills assessment with instructional feedback in ultrasound-guided interventions. Int J Comput Assist Radiol Surg. 2019;14(11):1993–2003. 31006107 10.1007/s11548-019-01977-3

[67] Hashmi OU, Chan N, de Vries CF, Gangi A, Jehanli L, Lip G. Artificial intelligence in radiology: trainees want more. Clin Radiol. 2023;78(4):e336–41. 36746724 10.1016/j.crad.2022.12.017

